# Effect of Caffeine on the Inflammatory-Dependent Changes in the GnRH/LH Secretion in a Female Sheep Model

**DOI:** 10.3390/ijms25052663

**Published:** 2024-02-25

**Authors:** Andrzej Przemysław Herman, Monika Tomczyk, Maciej Wójcik, Joanna Bochenek, Hanna Antushevich, Anna Herman, Wiktoria Wiechetek, Aleksandra Szczepkowska, Elżbieta Marciniak, Dorota Tomaszewska-Zaremba

**Affiliations:** 1 The Kielanowski Institute of Animal Physiology and Nutrition, Polish Academy of Sciences, 05-110 Jabłonna, Poland; m.tomczyk@ifzz.pl (M.T.); m.wojcik@ifzz.pl (M.W.); j.bochenek@ifzz.pl (J.B.); a.antuszewicz@ifzz.pl (H.A.); w.wiechetek@ifzz.pl (W.W.); e.marciniak@ifzz.pl (E.M.); d.tomaszewska@ifzz.pl (D.T.-Z.); 2 Chair of Drug and Cosmetics Biotechnology, Faculty of Chemistry, Warsaw University of Technology, 00-662 Warsaw, Poland; anna.herman@pw.edu.pl; 3 Department of Ichthyology and Biotechnology in Aquaculture, Institute of Animal Sciences, University of Life Sciences, 02-786 Warsaw, Poland; 4 Institute of Animal Reproduction and Food Research, Polish Academy of Sciences, 10-748 Olsztyn, Poland; a.szczepkowska@pan.olsztyn.pl

**Keywords:** caffeine, inflammation, GnRH, luteinizing hormone, hypothalamus, pituitary

## Abstract

Caffeine is one of the most widely consumed psychoactive drugs in the world. It easily crosses the blood–brain barrier, and caffeine-interacting adenosine and ryanodine receptors are distributed in various areas of the brain, including the hypothalamus and pituitary. Caffeine intake may have an impact on reproductive and immune function. Therefore, in the present study performed on the ewe model, we decided to investigate the effect of peripheral administration of caffeine (30 mg/kg) on the secretory activity of the hypothalamic–pituitary unit which regulates the reproductive function in females during both a physiological state and an immune/inflammatory challenge induced by lipopolysaccharide (LPS; 400 ng/kg) injection. It was found that caffeine stimulated (*p* < 0.01) the biosynthesis of gonadotropin-releasing hormone (GnRH) in the hypothalamus of ewe under both physiological and inflammatory conditions. Caffeine also increased (*p* < 0.05) luteinizing hormone (LH) secretion in ewes in a physiological state; however, a single administration of caffeine failed to completely release the LH secretion from the inhibitory influence of inflammation. This could result from the decreased expression of GnRHR in the pituitary and it may also be associated with the changes in the concentration of neurotransmitters in the median eminence (ME) where GnRH neuron terminals are located. Caffeine and LPS increased (*p* < 0.05) dopamine in the ME which may explain the inhibition of GnRH release. Caffeine treatment also increased (*p* < 0.01) cortisol release, and this stimulatory effect was particularly evident in sheep under immunological stress. Our studies suggest that caffeine affects the secretory activity of the hypothalamic–pituitary unit, although its effect appears to be partially dependent on the animal’s immune status.

## 1. Introduction

The complex interaction between the endocrine and immune systems may result in the emergence of endocrine disorders. The endocrine glands may be directly involved in infectious and inflammatory processes in the course of systemic inflammation. Infectious and inflammatory disorders of endocrine tissues may lead in turn to a disturbance in hormone production and activity [1]. One of the endocrine systems sensitive to inflammation is the hypothalamic–pituitary–gonadal (HPG) axis, which controls the reproductive process in female mammals. It is well known that inflammatory processes may affect female fertility [2]. On the one hand, several inflammatory mediators contribute to paracrine and endocrine signaling and the maintenance of tissue homeostasis in the female reproductive tract. On the other hand, inflammation caused by viral or bacterial infection may disturb the secretory activity of the hypothalamus and pituitary, cause endometriosis, and perturb ovarian follicle development [3,4]. Our previous study performed on the sheep model showed that inflammation induced by the administration of bacterial endotoxin–lipopolysaccharide (LPS) suppresses the synthesis of gonadotropin-releasing hormone (GnRH) in the hypothalamus and the secretion of luteinizing hormone (LH) in the pituitary [5,6] and that inflammatory cytokines, particularly interleukin (IL)-1β, play an important role in the inflammatory-dependent suppression of GnRH/LH secretion [7,8]. It is worth mentioning that the inhibitory action of proinflammatory cytokines on GnRH at the level of the hypothalamus may involve different neural mechanisms: direct action on the GnRH neurons through their corresponding receptor as well as indirect action involving other mediators such as opioids, catecholamines, gamma-aminobutyric acid, prostaglandins, or nitric oxide [9]. Moreover, we showed that the pharmacological inhibition of the production of these cytokines may diminish the suppression of GnRH/LH secretion, which accompanies systemic inflammation [5,8].

However, currently available anti-inflammatories are not completely reliable and/or cause intolerable side effects. Therefore, new anti-inflammatory substances are still under examination. Plants were the first source of remedies in the history of mankind, and herbal bioactive compounds have fueled drug development. Currently, the knowledge considering the usability of plant-derived pharmacologically active agents, in particular that of anti-inflammatory compounds, is continuously increasing [10]. One of the plant-delivered compounds that have been known for years, but are currently discovering new possibilities for their use in medicine, is caffeine. Caffeine, a compound classified in the methylxanthine class, is the most widely consumed psychoactive drug in the world. Natural sources of caffeine include coffee, tea, and chocolate. Synthetic caffeine is also added to products to promote arousal, alertness, energy, and elevated mood [11]. Caffeine acts as a stimulant, antioxidant, anti-inflammatory, and even an aid in pain management, and is found in several over-the-counter medications. Currently, caffeine’s effect on cancer and cardiovascular, immunological, inflammatory, and neurological diseases is intensively studied [12].

Caffeine receptors are widespread in the immune cells, and due to that it can affect their secretory activity and the course of the inflammatory response [13]. Moreover, the expression of genes encoding caffeine-interacting adenosine receptors (ADORs) and ryanodine receptors (RYRs) was found in the hypothalamic structures involved in the GnRH secretion and the anterior pituitary (AP) [14].

Therefore, in the present study, we decided to investigate the effect of peripheral administration of this methylxanthine on the secretion of GnRH and LH in the ewes during the follicular phase of the estrous cycle and to determine whether inflammation induced by LPS injection modulates the response of hypothalamic–pituitary unit to caffeine action.

## 2. Results

### 2.1. Effect of Caffeine and LPS Administration on the Circulating Concentration of LH, FSH, Estradiol, and Cortisol

**Figure 1:**
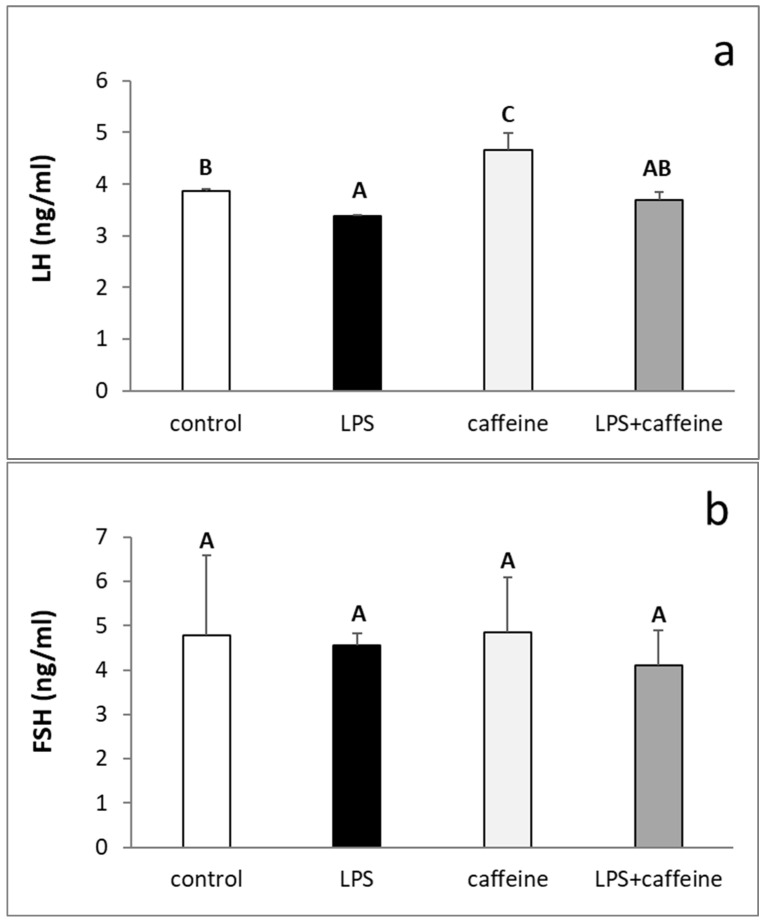
Effect of lipopolysaccharide (LPS; 400 ng/kg; iv.) and caffeine (30 mg/kg; iv.) injections on the concentration of luteinizing hormone (LH) (**a**) and follicle-stimulating hormone (FSH) (**b**) in the blood plasma. The data are presented as the mean value ± S.E.M. (*n* = 6 animals per group) of hormone concentration assayed during a period after the treatment (2 to 3 h after). Significant differences marked with different capital letters were analyzed by a two-way ANOVA followed by a Fisher’s post hoc test. Statistical significance was stated when *p* < 0.05.

**Figure 2:**
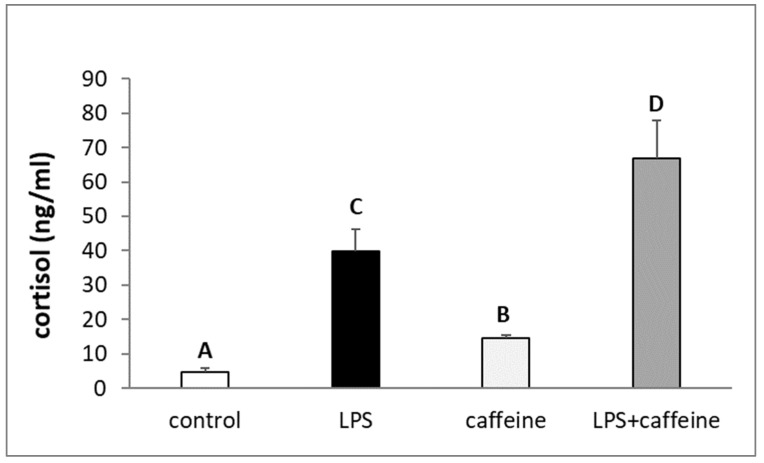
Effect of lipopolysaccharide (LPS; 400 ng/kg; iv.) and caffeine (30 mg/kg; iv.) injections on the concentration of cortisol in the blood plasma. The data are presented as the mean value ± S.E.M. (*n* = 6 animals per group) of hormone concentration assayed during a period after the treatment (2 to 3 h after). Significant differences marked with different capital letters were analyzed by a two-way ANOVA followed by a Fisher’s post hoc test. Statistical significance was stated when *p* < 0.05.

**Figure 3:**
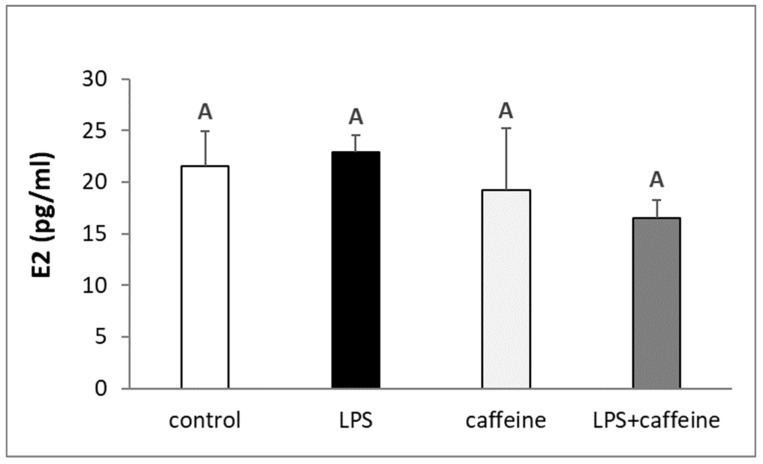
Effect of lipopolysaccharide (LPS; 400 ng/kg; iv.) and caffeine (30 mg/kg; iv.) injections on the concentration of estradiol (E2) in the blood plasma. The data are presented as the mean value ± S.E.M. (*n* = 6 animals per group) of hormone concentration assayed during a period after the treatment (2 to 3 h after). Significant differences marked with different capital letters were analyzed by a two-way ANOVA followed by a Fisher’s post hoc test. Statistical significance was stated when *p* < 0.05.

The injection of LPS reduced (*p* < 0.05) the circulating concentration of LH, whereas the peripheral administration of caffeine stimulated (*p* < 0.05) LH release. The plasma concentration of LH in animals treated with both LPS and caffeine did not differ from the concentration found in the LPS-treated animals, but also in the control group (Figure 1a). On the other hand, there was no effect of the experimental treatments on the blood level of FSH (Figure 1b). The administration of bacterial endotoxin stimulated (*p* < 0.05) cortisol release. The injection of caffeine also increased *p* < 0.05) the circulating level of cortisol in non-LPS treated ewes, but this increase was lower (*p* < 0.05) in comparison to the LPS-treated group. As indicated, in animals treated with both LPS and caffeine, the plasma concentration of cortisol was the highest (*p* < 0.05) among all experimental groups (Figure 2). Experimental treatments did not influence the blood concentration of estradiol (E2) in ewes (Figure 3).

### 2.2. Effect of Caffeine and LPS Administration on GnRH Content in the Preoptic Area of the Hypothalamus

**Figure 4:**
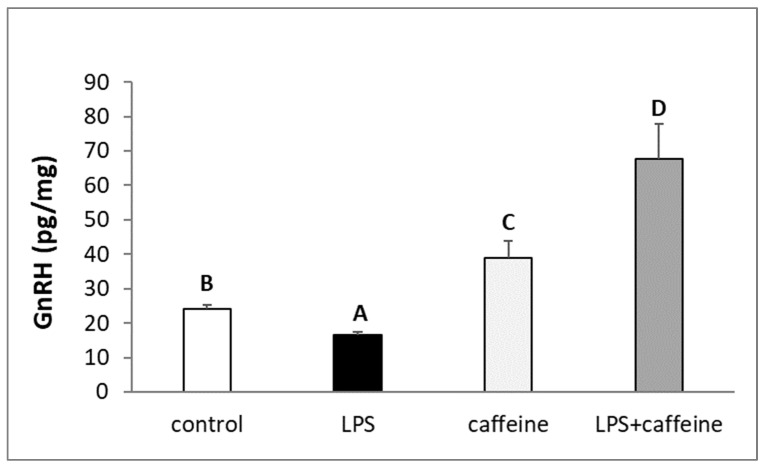
Effect of lipopolysaccharide (LPS; 400 ng/kg; iv.) and caffeine (30 mg/kg; iv.) injections on the concentration of gonadotropin-releasing hormone (GnRH) in the preoptic area of the hypothalamus. The data are presented as the mean value ± S.E.M. (*n* = 6 animals per group). Significant differences marked with different capital letters were analyzed by a two-way ANOVA followed by a Fisher’s post hoc test. Statistical significance was stated when *p* < 0.05.

It was found that the endotoxin treatment decreased (*p* < 0.05) the level of GnRH peptide in the POA. On the other hand, a single injection of caffeine increased the concentration of GnRH in this hypothalamic structure. In the POA of animals simultaneously injected with caffeine and LPS, the content of GnRH was the highest in comparison with all other experimental groups (Figure 4).

### 2.3. Effect of Caffeine and LPS Administration on the Concentration of Neurotransmitters Regulating the Release of GnRH and Their Metabolites in the Median Eminence

Peripherally administered caffeine increased (*p* < 0.05) the concentration of norepinephrine (NE), dopamine (DA), and serotonin (5-HT) in the ME, whereas endotoxin-induced acute inflammation increased (*p* < 0.05) the level of DA, homovanillic acid (HVA), and tryptophan (TRP) in this hypothalamic structure. In animals concomitantly treated with LPS and caffeine, the concentration of DA reached a higher level (*p* < 0.05) in comparison with other experimental groups. On the other hand, in these animals, the NE and 5-HT concentrations did not differ from those determined in the control group. In animals treated with LPS, caffeine administration did not influence the level of HVA and TRP in the ME, which was the same as in the case of the group injected only with LPS (Table 1).

### 2.4. Effect of Caffeine and LPS Administration on the Gene Expression in the Hypothalamus and Anterior Pituitary

Injection of caffeine increased (*p* < 0.05) the gene expression of *LHβ* in the AP of non-LPS treated ewes during the follicular phase of the estrous cycle. Acute inflammation included by bacterial endotoxin injection lowered (*p* < 0.05) the level of mRNA encoding *LHβ* and gonadotropin-releasing hormone receptor (*GnRHR*) in the AP. In the group of animals treated with both caffeine and LPS, the gene expression of *GnRHR* did not differ from those determined in the LPS-treated group, whereas the level of *LHβ* mRNA was the same as that in both the LPS-treated and control groups (Table 2).

Caffeine treatment increased (*p* < 0.05) the gene expression of *GnRH* in the preoptic area (POA) and ME. On the other hand, LPS injection reduced (*p* < 0.05) the amount of *GnRH* mRNA in these hypothalamic structures. In animals treated with both LPS and caffeine, the expression of *GnRH* mRNA in the POA and ME did not differ from those found in the control and caffeine-treated groups (Table 3).

## 3. Discussion

Our studies in a sheep model showed for the first time that the effect of caffeine on the hypothalamic–pituitary unit in ewes depends on the immune status of the animals. On the one hand, in a physiological state, intravenously administered caffeine stimulated the GnRH/LH secretion during the follicular phase of the estrous cycle. On the other hand, caffeine abolished the effect of LPS-induced inflammation on GnRH synthesis and even stimulated its synthesis by the hypothalamic structure POA [15]. However, a single injection of caffeine did not affect LH secretion which remained inhibited by LPS administration.

### 3.1. Effect of Caffeine on the GnRH/LH Secretion in Non-LPS Treated Ewes

This stimulatory effect of caffeine on the GnRH/LH secretion may be a bit surprising because it was formerly believed that caffeine consumption by a woman may disturb the reproduction process in females and extend the time it takes to become pregnant [16,17]. At the same time, a study on male rabbits analyzing the hormonal and histological effects of chronic caffeine administration on the pituitary–gonadal axis showed that caffeine stimulated FSH release and reduced LH secretion [18]. A more recent study on male Wistar rats also showed the implication of caffeine consumption on reproductive functions, since prolonged caffeine consumption resulted in a decrease in serum concentration of LH and FSH [19]. Moreover, extensive studies on rat models showed that maternal caffeine consumption affects the development of the reproductive system and has deleterious long-term effects on reproductive efficiency and fertility of male offspring in the peripubertal, postpubertal, and adulthood periods [20]. It is worth mentioning that the precise mechanism underlying caffeine-induced male reproductive toxicity is still elusive. However, the current prevailing opinion is that there is not enough convincing evidence to be certain that caffeine can cause fertility problems. The results of an extensive analysis of scientific data suggest that the intake of low, medium, and high doses of caffeine intake does not appear to induce reproduction disorders in humans [21]. Our results obtained from ewes in a follicular phase showing the stimulatory effect of caffeine on LH secretion differs from the data obtained in an earlier study on oestradiol-implanted ovariectomized ewes, which showed that a single intravenous dose of caffeine did not affect circulating concentrations of gonadotropins but elevated the level of prolactin [22]. It could be assumed that these differences may result from different hormonal statuses of ewes because sometimes in ewes with estradiol implants, circulating concentrations of this hormone may reach a significantly higher level [23] in comparison to those determined in follicular phase ewes in our study. It was determined that estrogen may significantly reduce the rate of caffeine metabolism [24]. Although it could be assumed that the estrogen-dependent prolongation of the half-life of caffeine in the organism can lead to the intensification of its biological effects, it is currently suggested that there is rather an inverse relationship between the action of caffeine and the level of estrogens. It is believed that ovarian steroids reduce or even inhibit many of the physiological effects of caffeine in the organism of females [25]. It is worth mentioning that the estrogen–caffeine interaction seems to be more complex and ambiguous because one study suggested that the consumption of caffeine may increase the estradiol level in women [26]; however, other work reported that moderate consumption of caffeine was associated with reduced estradiol concentrations among white women [27]. However, our study suggests that the acute administration of caffeine did not influence the circulating level of this hormone in ewes.

Although our previous ex vivo experiment suggests that caffeine may directly influence the secretory activity of the AP and stimulate the biosynthesis and release of LH [28], it rather appears that in the present in vivo experiment, caffeine influences LH release at the hypothalamic level by modulating GnRH secretion in the hypothalamus. Caffeine is water- and fat-soluble so it can easily cross the blood–brain barrier (BBB). Caffeine was found to enter the brain via both simple diffusion and saturable, carrier-mediated transport [29] and may even modulate this barrier permeability [30]. As mentioned above, *ADORs* and *RYRs* mRNA were found right in the hypothalamic structures involved in the GnRH secretion [14], which indicates that caffeine has a direct effect on these areas of the hypothalamus. We found that the peripheral administration of caffeine stimulated the biosynthesis of GnRH in the POA and increased the accumulation of mRNA for *GnRH* in the ME. The increased secretion of GnRH in the hypothalamus may cause the same effect in LH release. This supports the results of the previous study which showed that the facilitation of Ca^2+^ release from the intracellular store by caffeine increased the spontaneous release as well as evoked release of GnRH in bullfrog sympathetic ganglia [31].

### 3.2. Influence of Caffeine on the Synthesis of Neurotransmitters Involved in the Regulation of GnRH Secretion in the Hypothalamus

Caffeine may also influence GnRH secretion indirectly via modulation of the secretion of numerous neurotransmitters in the hypothalamus. Our experiment showed that injection of caffeine in ewes in a physiological state increased the concentration of NE, DA, and 5-HT in the ME—the hypothalamic structure where the majority of GnRH neurons’ terminals are located and where this neurohormone is released into the hypophyseal portal system. NE is considered to be one of the most important neurotransmitters involved in the regulation of GnRH secretion. The results of the study on GnRH-secreting immortalized hypothalamic (GT1-7) neurons showed that NE directly affects the membrane potential of GT1-7 cells via β-adrenergic receptors and induces Ca^2+^ mobilization, which in turn results in the stimulation of GnRH secretion [32]. On the other hand, DA is considered to be one of the most potent inhibitors of GnRH neuron excitability. A study on male and female mice showed that this inhibition is achieved through complex pre- and postsynaptic actions that involve D1- and D2-like receptor activation [33]. Moreover, a study conducted on sheep suggested the inhibitory role of dopaminergic neurotransmission through the D2 receptor on the regulatory pathways of GnRH biosynthesis in the hypothalamic–pituitary unit [34]. The role of 5-HT in the regulation of the secretory activity is more elusive. An in vitro study on hypothalamic GT1–7 cells showed that serotonin stimulated the release of GnRH through the Src-PLC γ1 pathway via the modulation of intracellular calcium levels [35]. However, more recent studies suggest that the role of 5-HT in the regulation of GnRH neurons is more complex and that 5-HT exerts a biphasic action on most GnRH neurons whereby a fast 5HT(1A)-mediated inhibition occurs alongside a slow 5-HT(2A) excitation. The balance of 5-HT-evoked inhibition vs. excitation is developmentally regulated, sexually differentiated, and variable across the estrous cycle [36]. In conclusion, the action of caffeine on the GnRH-modulating neurotransmitters is not completely clear because it activates both stimulatory and inhibitory pathways in the hypothalamus. Therefore, it seems that the effect that caffeine exerts on the GnRH-ergic neurons is the result of a dynamic balance between these mechanisms.

### 3.3. Possible Involvement of Cortisol in Caffeine-Mediated Effect on GnRH/LH Secretion

Our results also showed that the acute administration of caffeine significantly elevated the circulating level of cortisol in ewes. The correlation between caffeine consumption and the increased circulating concentration of cortisol has been previously reported in women and men; however, some sex-related differences in the cortisol response were found [37,38]. Stress-like elevations in circulating glucocorticoids are considered to suppress GnRH and gonadotropin secretion. It was demonstrated that in sheep, cortisol acts at the pituitary to reduce its responsiveness to GnRH and LH pulse frequency as well as suppresses GnRH pulse frequency in follicular phase ewes, and this appears to be dependent upon the presence of ovarian steroids [39]. It is generally accepted that stress has a generally inhibitory effect on GnRH/LH secretion. The deleterious effects of stress exposure on reproductive function have been demonstrated in several species, with a majority of neuroendocrine studies in primates, sheep, and rodents [40]. However, a growing body of evidence suggests that the role of stress in the modulation of gonadotropin secretion is more ambiguous. Recent works suggest that acute stress does not inhibit but instead stimulates the activity of the hypothalamic–pituitary unit. A study on rats showed that acute stress anticipates and amplifies the pre-ovulatory surge of LH [41]. It was also reported that in some cases, stress exerts stimulatory effects on reproductive neuroendocrine function. It is suggested that activation of corticotropin-releasing hormone (CRH) receptors, in particular CRHR1, can enhance LH secretion under certain conditions [40]. The authors further indicate that the high level of estradiol, typical for the late follicular/proestrus phase and consistent with our studies, may prepare the reproductive neuroendocrine system to respond to stress through activation [40].

### 3.4. The Effect of Inflammation on the Responsiveness of the Hypothalamic–Pituitary Unit to Caffeine Action

The study showed that on the one hand inflammation influences the caffeine-mediated changes in the GnRH/H secretion, on the other hand, caffeine administration may abolish some negative effects of inflammation on the secretory activity of the hypothalamic–pituitary unit. In contrast to animals in the physiological state, caffeine administration did not stimulate LH secretion in LPS-treated ewes. However, it should be pointed out, that the gene expression of LHβ in the AP as well as the circulating level of LH found in animals treated with both endotoxin and caffeine did not differ from those determined in both intact and single LPS-treated animals. This observation may indicate a slight pro-gonadotropic action of caffeine during acute inflammation, whereas a much stronger and clearer effect of caffeine was found at the level of the hypothalamus. We determined that similar to ewes in physiological state, caffeine stimulated GnRH mRNA expression in the POA and ME but caffeine-induced increase in the biosynthesis of GnRH peptide was significantly higher in LPS-treated individuals in comparison to ewes injected only with caffeine. The reason for this phenomenon is not entirely clear. In a previous study on ewes, we did not determine the pattern of changes in the gene expression of caffeine-interacting receptors in *ADORs* and *RYRs* receptors, which could suggest that LPS-treated ewes are more sensitive to caffeine action at the level of the hypothalamus [14]. However, the results of our previous study showed that caffeine may downregulate the synthesis of some pro-inflammatory cytokines in the hypothalamus which were identified as inhibitors of GnRH secretion [42]. This study showed that the expression of TNFα in ewes treated with both LPS and caffeine was lower in comparison with not only the LPS-treated group but even with intact animals. This result is not a surprise because many studies highlight the inhibitory effects of caffeine on TNFα production [43,44]. Besides IL-1β, TNFα is considered one of the most potent suppressors of GnRH secretion involved in the transmission of inflammatory effects on the function of GnRH-ergic neurons [45,46]. Therefore, the reduction in the local synthesis of TNFα by caffeine may have contributed to the increased synthesis of GnRH. The fact that the increase in biosynthesis of GnRH did not correlate with increased secretion of LH may at least partially result from the changes in the neurotransmitters in the ME. In ewes injected with both LPS and caffeine, a significant increase in the concentration of DA was found, and as stated above, DA is one of the main inhibitors of GnRH release [33,34]. This increase in DA content in the ME was accompanied by increased DA turnover because of a significant increase in the concentration of the main dopamine metabolite-homovanillic acid (HVA) in ewes concomitantly treated with LPS and caffeine. This increase could result from the stress caused by an immune inflammatory challenge because stress is known to cause an increase in dopamine metabolism and release [47,48]. The same increased concentration of tryptophan in the ME could result as an effect of the acute stress induced by endotoxin injection. Acute stress increases brain tryptophan levels through the enhancement of sympathetic nervous system activity. In turn, increased tryptophan hydroxylase activity in stress further influences glucocorticoid response [49]. It is worth pointing out that our study showed that the level of circulating cortisol—one of the stress markers in the ewes treated with both LPS and caffeine—was the highest among all experimental groups. This strong elevation of circulating cortisol levels in these animals suggests that immune stress and caffeine increase the cortisol release from the adrenal gland via completely or partially independent mechanisms; however, it requires further detailed research. Finally, the fact that increased GnRH synthesis did not result in the increased release of LH could result from the decreased sensitivity of the AP on the GnRH action. One of the mechanisms via inflammation to reduce LH secretion is the reduction in the expression of GnRHR in the AP, which results in decreased responsiveness of this gland on GnRH action [8,50].

### 3.5. Pharmacokinetics of Caffeine

It is worth mentioning that some of the observed effects of caffeine may be caused by circulating caffeine metabolites. Caffeine is metabolized in the liver by the cytochrome P-450 enzyme system to the main product paraxanthine and the additional products theobromine and theophylline [51]. They exhibit numerous biological activities. Paraxanthine has been shown to have several of the same nootropic benefits as caffeine, including improved cognition, short-term memory, sustained attention in healthy adults, and beneficial cognitive effects in animal models of Parkinson’s disease [52]. Paraxanthine exhibits higher than caffeine-binding potencies for adenosine A1 and A2A receptors in the forebrain and has a stronger locomotor activating effect leading paraxanthine to also function as a central nervous stimulant like caffeine [53]. It was also found to suppress neutrophil and monocyte chemotaxis, and also inhibit the production of TNFα in human blood [54]. In turn, theobromine is involved in a large variety of brain processes, including the induction of the brain-derived neurotrophic factor which supports cell survival and neuronal functions, including learning and memory [55]. Theobromine has been shown to exhibit anti-inflammatory effects and reduce blood pressure and LDL cholesterol levels [56]. Theophylline is a nonselective adenosine receptor A1, A2, and A3 antagonist and it acts as a competitive nonselective phosphodiesterase inhibitor, which increases the concentration of intracellular cAMP and activates protein kinase A [57,58]. It was also shown to have some anti-inflammatory properties. Theophylline inhibits the synthesis of interferon-gamma and TNFα and stimulates IL10 production [59]. However, it is worth mentioning that the dynamic of the caffeine metabolism is species-dependent. In healthy humans, the average half-life of caffeine is 5 h, but in studies conducted on ovariectomized ewe, the half-life of caffeine was defined as 47 h [60]. Therefore, it can be assumed that in our studies performed on a sheep model, which was conducted for 3 h after a single administration of caffeine, the vast majority of the observed effects result from the direct impact of this methylxanthine.

## 4. Materials and Methods

### 4.1. Animals and Experimental Design

The experiment was performed on 2-year-old Blackhead ewes (*n* = 24) in the follicular phase of the estrous cycle during the breeding season (October). To standardize the experimental conditions, the stages of the estrous cycle of ewes were synchronized by the Chronogest^®^ CR (Merck Animal Health, Rahway, NJ, USA) method as was previously described [61]. The experimental procedures were performed 24 h following PMSG injection. On the morning of the experiment, ewes were placed in individual cages and randomly assigned into four experimental groups (Table 4) receiving intravenously the following treatment: (1) control group—double saline (0.9% *w*/*v* NaCl); (2) LPS group—LPS (*Escherichia coli* 055:B5, Merck KGaA, Darmstadt, Germany) in a dose of 400 ng/kg to induce immune stress, which was established based on our previous research [5,8,14], and then saline; (3) caffeine group—saline and then caffeine (Merck KGaA, Darmstadt, Germany) in a dose of 30 mg/kg chosen based on a previous study [42]; and (4) LPS + caffeine group—LPS and then caffeine. During the experiment, the body temperature was measured and blood samples were collected through a catheter inserted into the jugular vein starting 2 h before administration of the experimental agents and then for another 3 h. The ewes were sacrificed 3 h after treatment and after decapitation, the brains were rapidly removed from the skulls and the hypothalamus and AP were dissected and at once frozen in liquid nitrogen.

### 4.2. Assays

#### 4.2.1. Radioimmunoassay of Hormones

The concentration of LH in blood plasma was assayed with a double-antibody radioimmunoassay (RIA) using the protocol described by Stupnicki and Madej [62]. The plasma FSH concentration was determined by double antibody RIA according to L’Hermite et al. [63]. The concentration of cortisol was assayed according to the Kokot and Stupnicki method [64].

#### 4.2.2. ELISA Assay for the GnRH and Estradiol

The concentrations of GnRH in the POA homogenate prepared as previously described [8] were determined using a commercial GnRH ELISA kit (cat. no. ESH0062, FineTest Biotech Inc., Boulder, CO, USA) appropriate for sheep. The values of GnRH concentration were normalized to the total protein content in each sample assayed using the Bradford method [65]. The concentrations of estradiol in the blood plasma were determined using a commercial estradiol ELISA kit (cat. no. EA0018Sh; Shanghai Korain Biotech, Shanghai, China) designed for sheep.

#### 4.2.3. HPLC Assays for Neurotransmitters and Their Metabolites

The concentration of neurotransmitters and their metabolites in the ME was determined using high-performance liquid chromatography (HPLC) with electrochemical detection using Thermo Scientific Ultimate 3000HPLC system with Thermo Scientific Chromelon 7 software equipped with the LC-18-DB (15 cm × 4.6 mm IDx 5 µm) column, and Thermo Scientific “Dinex” Test Phase, according to a previous method described elsewhere [66].

#### 4.2.4. Relative Gene Expression Assay

Relative gene expression assays were performed by two-step RT-qPCR according to a previously published method [67]. NucleoSpin^®^ RNA kit (Macherey-Nagel GmbH and Co, Düren, Germany) was used to isolate the total RNA from the hypothalamic and AP samples. The purity and concentration of isolated RNA were quantified by measuring the optical density at 230, 260, and 280 nm using a NanoDrop 1000 (Thermo Fisher Scientific Inc., Waltham, MA, USA). The test of RNA integrity was carried out by electrophoretic analysis. cDNA synthesis was performed using 1 µg of total RNA and appropriate components of Maxima™ First Strand cDNA Synthesis Kit for RT-qPCR (Thermo Fisher Scientific Inc., Waltham, MA, USA). RT-qPCR was performed with the use of the HOT FIREPol EvaGreen^®^ qPCR Mix Plus (Solis BioDyne, Tartu, Estonia) and specific HPLC-grade oligonucleotide primers (Genomed, Warszawa, Poland) designed to determine the expression of the tested and reference genes (Table 5). One reaction mixture of total volume amounting to 15 µL contained 3 µL of PCR Master Mix, 10.05 μL of RNase-free water, 0.45 µL of primers (0.225 µL each primer), and 1.5 μL of the cDNA template. Real-time PCR reactions were carried out using Rotor-Gene Q (Qiagen, Dusseldorf, Germany) according to the following protocol: 95 °C for 15 min and 30–35 cycles at 95 °C for 10 s (denaturation), 59 °C for 20 s (primer annealing), and 72 °C for 10 s (extension). To verify the specificity of the PCR amplification, a final melting curve analysis and agarose gel electrophoresis of PCR products were carried out. The relative gene expression was calculated using the comparative quantification option of the Rotor Gene Q Series 2.1 Software (Qiagen, Dusseldorf, Germany) and normalized to the mean expression of three housekeeping genes: glyceralde-hyde-3-phosphate dehydrogenase (*GAPDH*), β-actin (*ACTB*), and histone deacetylase1 (*HDAC1*).

### 4.3. Statistical Analysis

Gene expression data were normalized to the average relative level of determined mRNA in the control group, which was set to 1.0. The statistical analysis was performed using the STATISTICA 10 software (Stat Soft. Inc., Tulsa, OK, USA). The analyses were performed on raw data after verification of normality assumptions (Shapiro–Wilk’s test). Data that failed the normality test were subjected to logarithmic transformation for further analysis. The results were analyzed using one-way analyses of variances (ANOVA) and followed by a post hoc Fisher’s least significance test. In figures and tables, different capital letters (A, B, C) indicate significant differences at *p* < 0.05, according to one-way ANOVA followed by Fisher’s post hoc test comparing groups with each other. Groups that do not differ statistically are marked with the same capital letter, while groups marked with different capital letters differ significantly. All results are presented as the mean ± SEM.

## 5. Conclusions

In summary, our study shows that the effect of caffeine on the activity of the hypothalamic–pituitary unit during immune stress is differentiated. On the one hand, caffeine can abolish the inflammatory-dependent suppression of GnRH biosynthesis in the hypothalamus; on the other hand, it failed to completely diminish the negative effect of inflammation on the LH secretion in the pituitary. It seems, however, that despite various and sometimes contradictory reports regarding the effects of caffeine on the HPG axis, our study suggests that the single and acute administration of this metaxanthine exerts rather a positive effect on the GnRH/LH secretion, particularly in animals at a physiological state. However, a detailed understanding of the potential role of caffeine in modulating the activity of the HPG axis in various physiological states requires further detailed research, especially in the context of long-term caffeine administration.

## Figures and Tables

**Table 1:** The effect of caffeine on the concentration of neurotransmitters and their metabolites in the median eminence.

	Control	LPS	Caffeine	LPS + Caffeine
Norepinephrine (NE)	6.24 ± 0.93 ^A^	8.85 ± 1.52 ^A^	18.30 ± 4.87 ^B^	14.03 ± 3.10 ^AB^
Dopamine (DA)	13.60 ± 1.65 ^A^	22.17 ± 2.74 ^B^	23.00 ± 3.00 ^B^	35.54 ± 5.08 ^C^
5-Hydroxyindoleacetic Acid (5-HIAA)	0.66 ± 0.13 ^A^	1.09 ± 0.11 ^A^	1.11 ± 0.29 ^A^	1.12 ± 0.19 ^A^
Homovanillic acid (HVA)	1.63 ± 0.14 ^A^	3.05 ± 0.26 ^B^	1.75 ± 0.25 ^A^	2.67 ± 0.49 ^B^
Serotonin (5-HT)	0.35 ± 0.06 ^A^	0.37 ± 0.03 ^A^	0.72 ± 0.09 ^B^	0.43 ± 0.03 ^A^
Tryptophan (TRP)	33.45 ± 2.45 ^A^	64.64 ± 2.63 ^B^	30.44 ± 5.80 ^A^	69.88 ± 8.40 ^B^

Legend: control—double treated with saline (0.9% NaCl iv.); LPS—treated with lipopolysaccharide (LPS; 400 ng/kg, iv.) followed by saline; caffeine—treated with saline followed by caffeine (30 mg/kg, iv.). All results are given in ng/mg of tissue. Different letters indicate significant differences at *p* < 0.05, according to one-way ANOVA followed by Fisher’s post hoc test comparing groups with each other.

**Table 2:** The effect of caffeine on the relative gene expression (mean ± SEM; *n* = 6) of *GnRH* receptor and beta subunit of gonadotropins (*LHβ, FSHβ*) in the anterior pituitary under basal and lipopolysaccharide challenge conditions.

Anterior Pituitary				
Gene	Control	LPS	Caffeine	LPS + Caffeine
*GnRHR*	1.00 ± 0.2 ^B^	0.44 ± 0.1 ^A^	0.74 ± 0.08 ^B^	0.49 ± 0.09 ^A^
*LHβ*	1.00 ± 0.09 ^B^	0.64 ± 0.04 ^A^	1.38 ± 0.11 ^C^	0.89 ± 0.07 ^AB^
*FSHβ*	1.00 ± 0.27 ^A^	0.91 ± 0.18 ^A^	1.18 ± 0.46 ^A^	1.13 ± 0.45 ^A^

Legend: control—double treated with saline (0.9% NaCl iv.); LPS—treated with lipopolysaccharide (LPS; 400 ng/kg, iv.) followed by saline; caffeine—treated with saline followed by caffeine (30 mg/kg, iv.); LPS + caffeine—treated with LPS followed by caffeine. GnRHR—gonadotropin-releasing hormone receptor; LHβ—luteinizing hormone subunit beta; FSHβ—follicle stimulating hormone subunit beta. Gene expression data were normalized to the average relative level of this mRNA expression in the control group, which was set to 1.0. Different letters indicate significant differences at *p* < 0.05, according to one-way ANOVA followed by Fisher’s post hoc test comparing groups with each other.

**Table 3:** The effect of caffeine on the relative gene expression (mean ± SEM; n = 6) of *GnRH* in the hypothalamus under basal and lipopolysaccharide challenge conditions.

*GnRH* Relative Gene Expression				
Hypothalamic Structure	Control	LPS	Caffeine	LPS + Caffeine
POA	1.00 ± 0.05 ^B^	0.76 ± 0.03 ^A^	1.11 ± 0.02 ^C^	1.30 ± 0.13 ^BC^
AHA	1.00 ± 0.35 ^A^	1.00 ± 0.31 ^A^	0.85 ± 0.20 ^A^	0.82 ± 0.28 ^A^
MBH	1.00 ± 0.28 ^A^	1.12 ± 0.41 ^A^	0.81 ± 0.25 ^A^	0.55 ± 0.15 ^A^
ME	1.00 ± 0.28 ^B^	0.30 ± 0.05 ^A^	1.98 ± 0.16 ^C^	1.48 ± 0.12 ^BC^

Legend: control—double treated with saline (0.9% NaCl iv.); LPS—treated with lipopolysaccharide (LPS; 400 ng/kg, iv.) followed by saline; caffeine—treated with saline followed by caffeine (30 mg/kg, iv.); LPS + caffeine—treated with LPS followed by caffeine. Hypothalamic structures: POA—preoptic area, AHA—anterior hypothalamus, MBH—medial basal hypothalamus, and ME—median eminence. Gene expression data were normalized to the average relative level of this mRNA expression in the control group, which was set to 1.0. Different letters indicate significant differences at *p* < 0.05, according to one-way ANOVA followed by Fisher’s post hoc test comparing groups with each other.

**Table 4:** Experiment organization chart.

Group	No. of Animals	Experimental Treatment I	Dose [ng/kg]	Experimental Treatment II	Dose [mg/kg]
control	6	NaCl	0	NaCl	0
LPS	6	LPS	400	NaCl	0
caffeine	6	NaCl	0	caffeine	30
LPS + caffeine	6	LPS	400	caffeine	30

**Table 5:** List of full names and abbreviations of all genes analyzed by RT-qPCR.

GenBank Acc. No.	Gene	Amplicon Size [bp]	Forward/Reverse	Sequence 5′ → 3′	Reference
NM_001034034	*GAPDH* *glyceraldehyde-3-phosphate dehydrogenase*	134	forward	AGAAGGCTGGGGCTCACT	[6]
			reverse	GGCATTGCTGACAATCTTGA	
U39357	*ACTB* *actin beta*	168	forward	CTTCCTTCCTGGGCATGG	[6]
			reverse	GGGCAGTGATCTCTTTCTGC	
BC108088.1	*HDAC1* *histone deacetylase1*	115	forward	CTGGGGACCTACGGGATATT	[8]
			reverse	GACATGACCGGCTTGAAAAT	
NM_001009397	*GnRHR* *gonadotropin-releasing hormone receptor*	150	forward	TCTTTGCTGGACCACAGTTAT	[6]
			reverse	GGCAGCTGAAGGTGAAAAAG	
U02517	*GnRH* *gonadotropin-releasing hormone*	123	forward	GCCCTGGAGGAAAGAGAAAT	[6]
			reverse	GAGGAGAATGGGACTGGTGA	
X52488	*LHB* *luteinizing hormone beta-subunit*	184	forward	AGATGCTCCAGGGACTGCT	[8]
			reverse	TGCTTCATGCTGAGGCAGTA	
X15493	*FSHB* *follicle-stimulating hormone beta-subunit*	131	forward	TATTGCTACACCCGGGACTT	[8]
			reverse	TACAGGGAGTCTGCATGGTG	

## Data Availability

The data presented in this study are available upon request from the corresponding author.
